# Fetal MRI: abdominal cystic lesions

**DOI:** 10.1186/s13244-025-02169-1

**Published:** 2026-01-28

**Authors:** David García Castellanos, Manuel Recio Rodríguez, Lucía Sanabria Greciano, Alejandra Aguado del Hoyo, Alejandro Díaz Moreno, Julia López Alcolea, Carolina Sampietro, Vicente Martínez de Vega

**Affiliations:** 1 Department of Radiology, Hospital Universitario Quirónsalud Madrid, Pozuelo de Alarcón, Madrid, Spain; 2 Department of Radiology, Hospital Universitario Gregorio Marañón, Madrid, Spain

**Keywords:** Magnetic resonance imaging, Fetal, Abdominal cysts, Prenatal diagnosis, Congenital abnormalities

## Abstract

**Abstract:**

Fetal MRI has become an essential tool for evaluating abdominal cystic lesions detected on prenatal ultrasound, offering superior soft tissue contrast and multiplanar imaging capabilities. This observational case series, conducted at Quironsalud Madrid University Hospital, analyzed fetuses diagnosed with abdominal cystic lesions who underwent fetal MRI. Lesions were classified into gastrointestinal, genitourinary, teratomatous, and syndromic categories. Fetal MRI allowed for precise lesion characterization, differentiating cystic masses from solid or mixed lesions, and identifying associated structural abnormalities. MRI findings were correlated with fetal ultrasound and, when available, postnatal imaging or surgical outcomes, demonstrating complementary information and improved diagnostic confidence compared to ultrasound alone. This improved accuracy has direct clinical implications, aiding in prenatal counseling, optimizing perinatal management, and guiding postnatal surgical planning. Our results reinforce the role of fetal MRI as a complementary imaging modality for refining the diagnosis of congenital abdominal cystic lesions and improving neonatal outcomes.

**Critical relevance statement:**

This article critically evaluates the role of fetal MRI, in conjunction with prenatal ultrasound, in characterizing abdominal cystic lesions, highlighting its diagnostic advantages over ultrasound and its clinical impact on prenatal counseling, perinatal management, and postnatal surgical planning in radiological practice.

**Key Points:**

- Abdominal cystic lesions are frequently detected on prenatal ultrasound, but their characterization and differentiation remain challenging.
- Fetal MRI characterizes lesions, assesses their extent, improves classification and diagnosis, and offers superior soft tissue contrast for evaluating complex anomalies.
- Fetal MRI complements prenatal ultrasound, allowing a more precise assessment of lesion characteristics and facilitating prenatal counseling and perinatal planning.

**Graphical Abstract:**

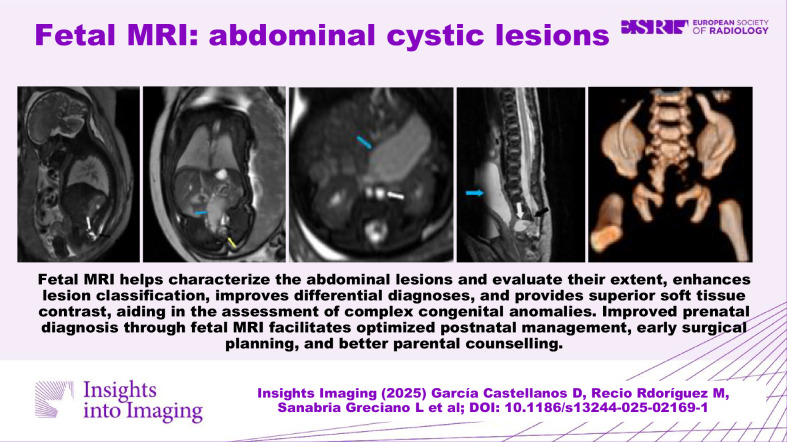

## Introduction

Fetal abdominal cystic lesions are commonly detected on prenatal imaging and encompass a wide range of differential diagnoses. The most frequent lesions include ovarian cysts (in female fetuses), choledochal cysts, hepatic, splenic, pancreatic, mesenteric, adrenal hemorrhagic, and renal cysts, as well as intestinal duplication cysts and meconium pseudocysts. Less common entities include cystic tumors such as neuroblastoma and intra-abdominal extralobar bronchopulmonary sequestration with cystic changes [1].

The detection of these cysts typically occurs in the second or third trimester, and isolated cysts identified earlier in gestation are often associated with favorable outcomes. Many fetal abdominal cysts originate from the gastrointestinal or genitourinary tract and may resolve spontaneously. However, establishing a precise diagnosis in utero remains challenging.

Magnetic resonance imaging (MRI) is used to identify and accurately classify these lesions, complementing prenatal ultrasound. using two natural contrast agents: swallowed amniotic fluid and fetal urine. Amniotic fluid, ingested from weeks 9-10, gradually enhances gastrointestinal visualization, while meconium fills the colon by week 27. Meanwhile, fetal urine, the primary source of amniotic fluid from week 10, aids in assessing the kidneys, bladder, and urinary structures.

In this review, we have analyzed the MRIs of multiple fetuses diagnosed with abdominal cystic lesions and classified them into four main groups: gastrointestinal cystic lesions, genitourinary cystic lesions, sacrococcygeal teratomas, and Currarino triad (Table 1).

**Table 1:** Classification of abdominal cystic lesions

GASTROINTESTINAL CYSTIC LESIONS	GENITOURINARY CYSTIC LESIONS	SACROCOCCYGEAL TERATOMA	CURRARINO TRIAD
- Intestinal duplication cyst - Intestinal dilation: duodenal, jejunal, or ileal atresia or stenosis, and intestinal volvulus - Meconium pseudocysts - Lymphatic malformation - Hepatic cyst - Hepatic cystic tumors - Biliary cyst - Splenic cyst	- Ovarian cyst - Hydrometrocolpos - Urogenital sinus - Cloacal malformation - Adrenal lesions: congenital adrenal cyst, adrenal hemorrhage (hemorrhagic pseudocysts), and cystic neuroblastoma - Renal lesions: multicystic dysplastic kidney, hydronephrosis, ureteropelvic junction stenosis, upper pole hydronephrosis in duplicated systems, and calyceal diverticulum - Megaureter - Bladder and/or ureteral dilation: posterior urethral valves, Eagle-Barrett syndrome, and hyperperistalsis syndrome (mega-bladder, microcolon, and intestine) - Urachal anomalies - Allantoic cyst - Omphalomesenteric cyst	- Type I - Type II - Type III - Type IV	- Sacrococcygeal defect - Presacral mass - Anorectal malformations

## Technical considerations

This case selection was conducted at Quiron Salud Madrid University Hospital, approved by the Institutional Review Board, with informed consent waived. It included fetuses diagnosed with abdominal cystic lesions on prenatal ultrasound who underwent fetal MRI between 2007 and 2024, using a GE Optima MR450w 1.5-T Scanner and a GE Premier MRI 3-T Scanner.

The sequences included depended on the case being studied but involved the following: T2-weighted (T2W) single-shot fast spin echo (SSFSE), Fast Imaging Employing Steady-State Acquisition (FIESTA), in-phase and out-of-phase gradient echo (GRE), T1-weighted (T1W), and diffusion-weighted imaging (DWI).

Cases with incomplete imaging or inconsistent postnatal diagnoses were excluded. Images were evaluated by an expert radiologist, categorizing lesions as gastrointestinal, genitourinary, cystic teratomas, or Currarino’s triad-related cystic lesions, with postnatal imaging or surgical findings used as the reference standard.

## Cystic lesions

### Gastrointestinal cystic lesions

#### Intestinal duplication cyst

Intestinal duplication cysts are rare congenital anomalies that can occur at various locations along the gastrointestinal tract. The most common site for these cysts is the ileum, accounting for approximately 34% of cases, followed by the colon, which represents about 18%. Duplication cysts in the stomach account for 2–7% of cases, and those in the distal esophagus are the least common [2]. Intestinal duplications typically occur along the mesenteric border, while gastric duplications are usually found along the greater curvature of the stomach.

Although intestinal duplication cysts are generally singular, multiple cysts are observed in approximately 5–7% of cases. These lesions typically present in two main forms: spherical (74–82%) or tubular (18–26%), with the majority being unilocular. It is important to note that the spherical type rarely communicates with the lumen, whereas the tubular type often does.

Polyhydramnios, which refers to an excessive amount of amniotic fluid, is uncommon in these cases unless the cyst causes gastrointestinal obstruction or if there is concurrent intestinal atresia.

About 50% of intestinal duplication cysts are associated with skeletal malformations. Additionally, one-third of midgut and hindgut duplications are associated with gastrointestinal or genitourinary anomalies, underscoring the importance of a thorough diagnostic and follow-up approach [2].

**Fig. 1:**
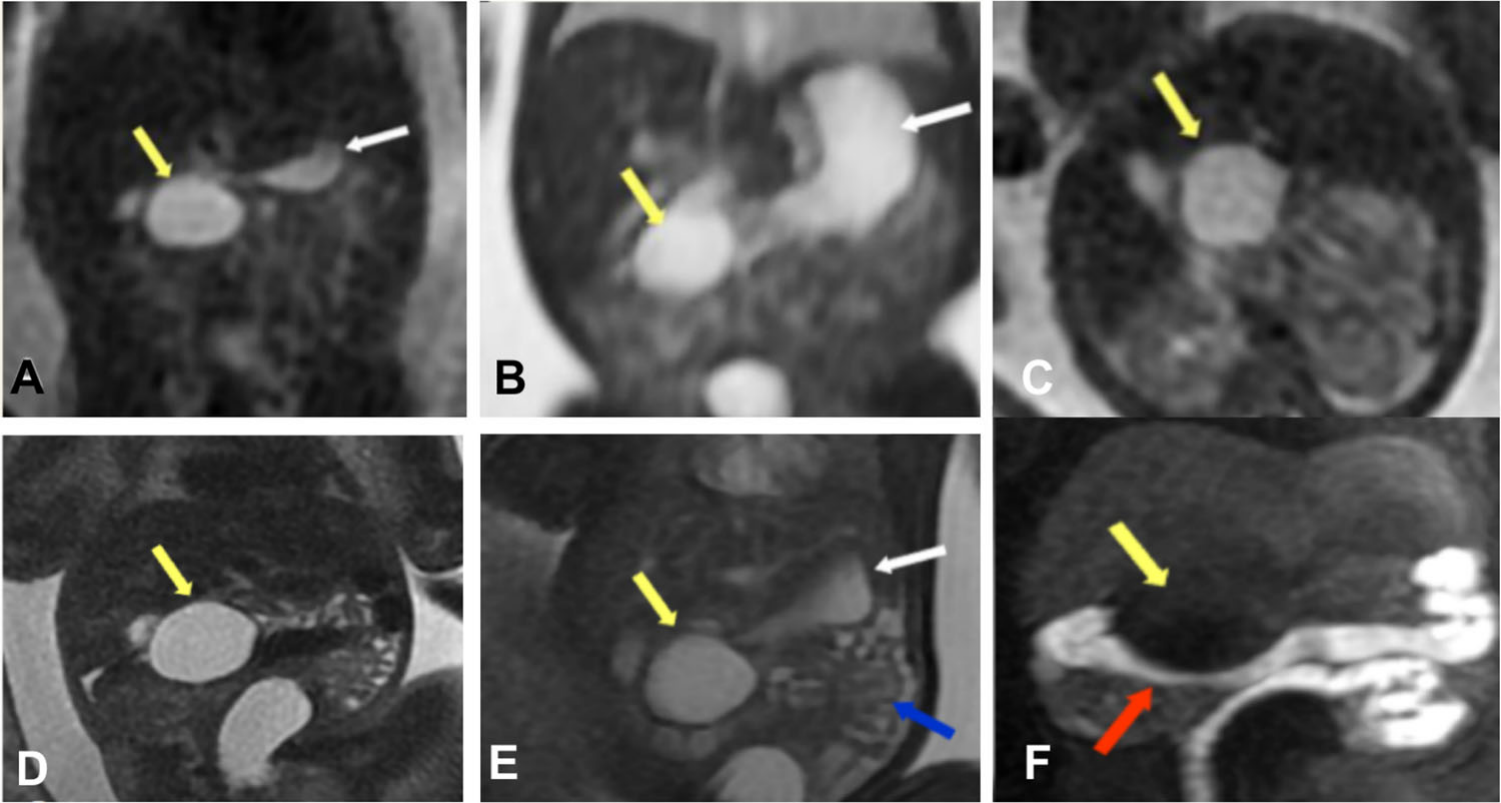
Intestinal Duplication Cyst. Fetal ultrasound showed a probable duodenal duplication cyst at a gestational age of 22 weeks and 1 day. **A** Coronal Single Shot FSE T2, (**B**) coronal FIESTA, and (**C**) axial SS FSE T2. Follow-up of the duodenal duplication cyst: (**D**) coronal SSFSE T2, (**E**) coronal FIESTA, and (**F**) coronal MIP 3D LAVA. The stomach is of normal size (white arrow), and the bowel loops are normal (blue arrow). The cyst indents the transverse colon (red arrows). A subhepatic cystic lesion is located anterior and medial to the second portion of the duodenum (yellow arrows). No connection with the stomach or the biliary tract is identified. MRI confirmed the ultrasound diagnosis

On MRI, most duplications appear with low signal intensity on T1W images and very high intensity on T2W images (Fig. 1) [2].

#### Duodenal atresia

Duodenal atresia is the most commonly detected intestinal atresia in the fetus. It is associated with several conditions, including annular pancreas, trisomy 21 (present in up to 30% of cases), preexisting diabetes, VACTERL association, and intestinal malrotation [3].

More than half of fetuses with duodenal atresia have associated anomalies, including cardiac, gastrointestinal (such as biliary atresia and agenesis of the gallbladder), renal, and vertebral abnormalities.

The differential diagnosis for duodenal atresia also includes annular pancreas, malrotation with Ladd’s bands, preduodenal portal vein, and choledochal cyst.

While ultrasound is usually sufficient to detect the classic “double bubble” sign, fetal MRI can offer added diagnostic value—particularly in distinguishing intrinsic causes like duodenal atresia from extrinsic compressive processes, such as malrotation with Ladd’s bands or annular pancreas. This distinction is clinically meaningful, as the prenatal identification of an extrinsic cause like malrotation may influence perinatal management decisions, including the place of delivery and the need for immediate neonatal surgical care. Therefore, MRI not only refines the diagnosis but may also guide obstetric planning and optimize neonatal outcomes.

**Fig. 2:**
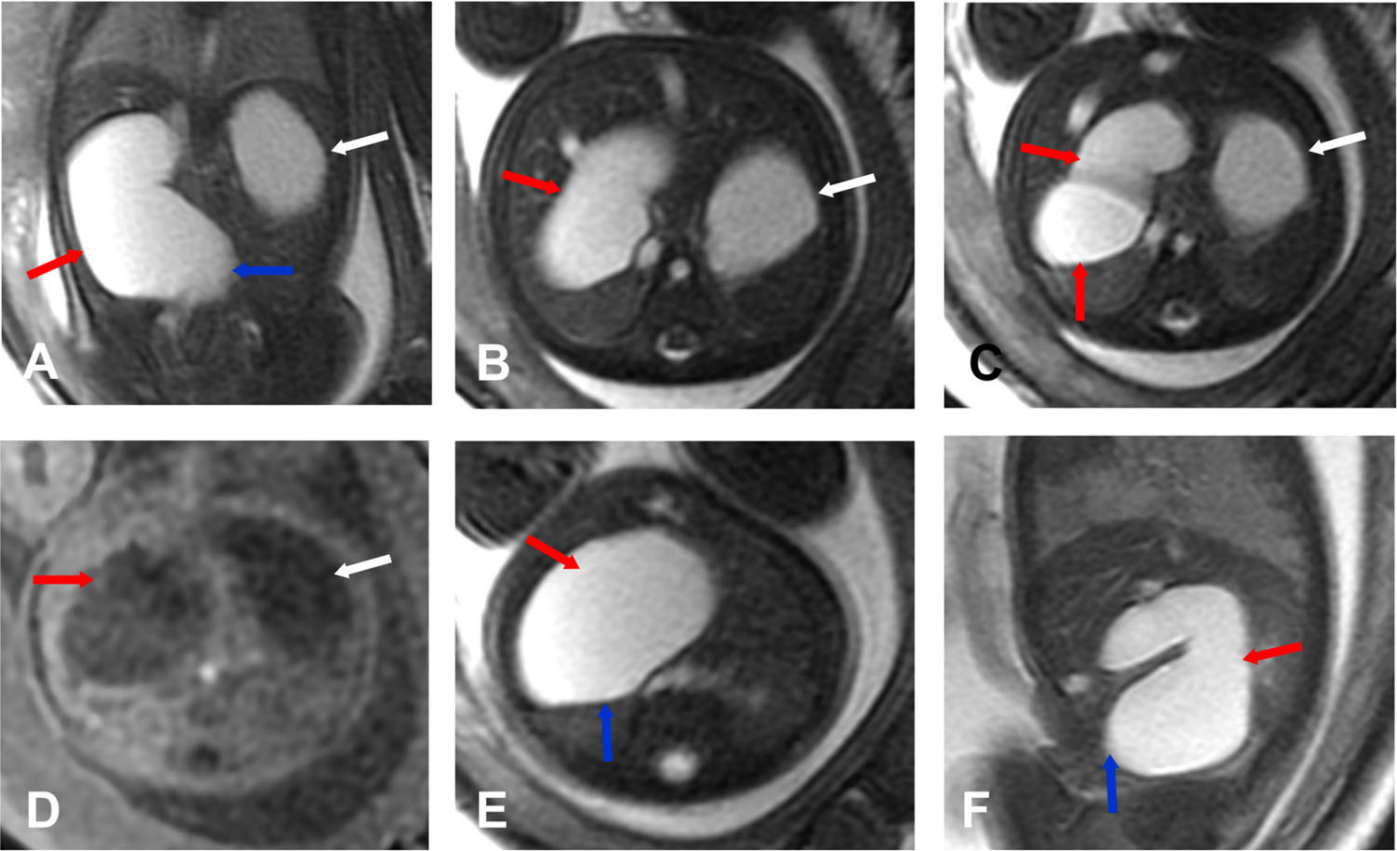
Duodenal atresia. Fetal ultrasound: duodenal atresia. Gestational age 30 weeks and 2 days. **A** Coronal FIESTA. **B**, **C** Axial FIESTA. **D** Axial 3D LAVA. **E** Axial FIESTA. **F** Sagittal FIESTA. Marked dilation of the stomach (white arrow) and duodenum (red arrow, demonstrating hypointense signal on T1 and hyperintense on T2 with the double bubble sign. Abrupt interruption of the lumen in the second duodenal bend due to atresia (blue arrow)

On MRI, T2W sequences typically reveal a dilated and fluid-filled stomach and proximal duodenum, presenting the classic “double bubble sign” without distal bowel dilation (Fig. 2). The communication between the cystic duodenal structure and the stomach helps differentiate duodenal atresia/stenosis from other conditions, such as a duplication cyst or choledochal cyst [3].

#### Jejunal atresia

**Fig. 3:**
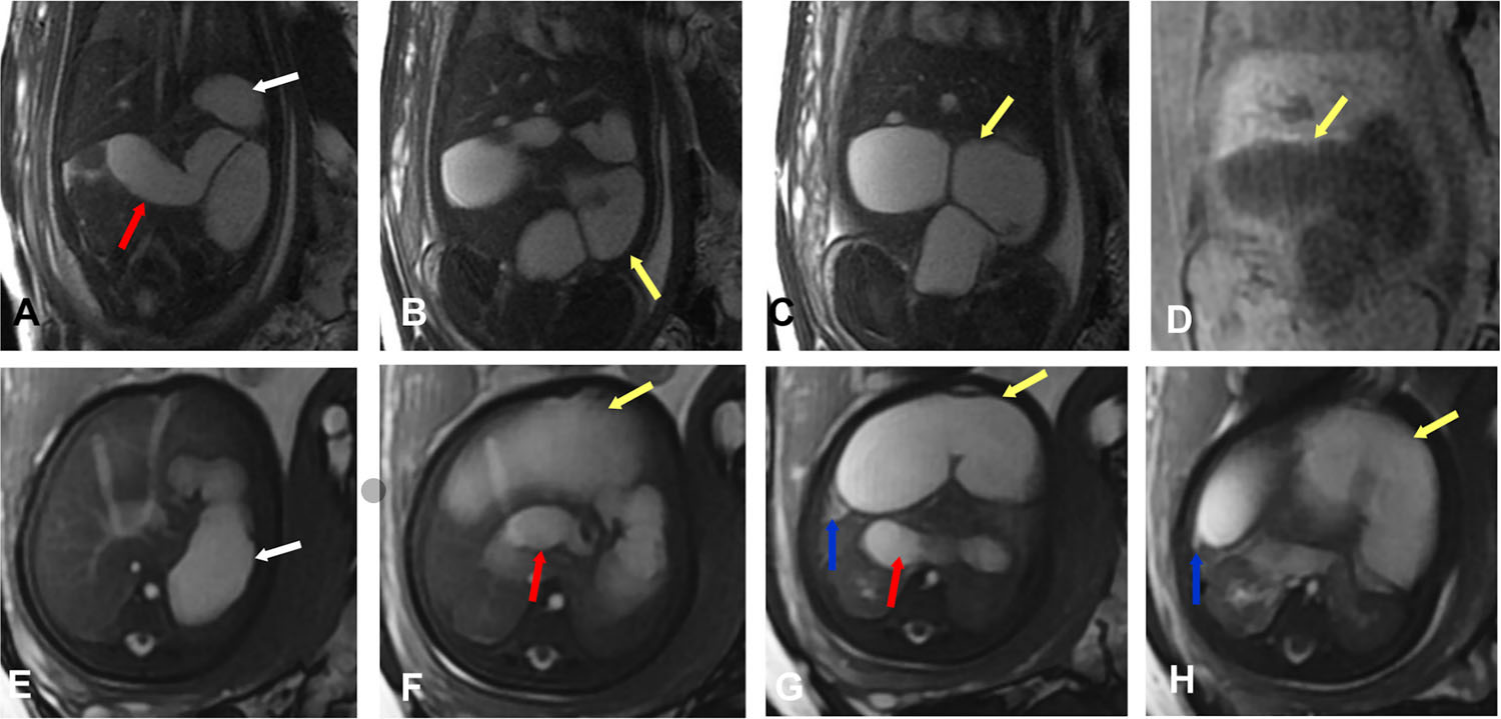
Jejunal atresia. Ultrasound: intestinal obstruction. Gestational age 32 weeks and 6 days. **A**, **B** Coronal FIESTA. **C** Coronal 3D LAVA. **D** Coronal FIESTA. **E**–**G** Axial FIESTA. **H** Axial cine FIESTA. Dilation of the stomach (white arrows), duodenum (red arrows), and especially the proximal jejunum (yellow arrows) up to the hepatic flexure, demonstrating hypointense signal on T1 and hyperintense on T2. A sudden change in caliber of the jejunal loop is identified at the hepatic flexure due to jejunal atresia (blue arrows)

Jejunal atresia accounts for 30% of intestinal atresia cases, with distal ileal atresia being more common (35%). Multiple atresias are seen in up to 6% of cases. It can be secondary to meconium ileus in 10% of cases (Fig. 3).

The incidence of associated aneuploidy and extraintestinal anomalies is low. However, common associated intestinal anomalies include gastroschisis, intestinal malrotation, and meconium ileus.

Children with jejunal atresia have a higher incidence of cystic fibrosis, which should be considered during diagnosis.

Jejunal atresia is classified into four types: Type 1 accounts for 32%, Type 2 for 25%, Type 3a for 15%, Type 3b for 11%, and Type 4 for 6% [4].

This classification helps guide treatment and predict outcomes for affected infants [5, 6] and [7].

#### Ileal atresia

MRI plays a crucial role in detecting the location of the obstruction in ileal atresia, often indicated by the number of dilated bowel loops.

The more proximal the obstruction, the higher the risk of polyhydramnios and intestinal perforation.

Jejunal and ileal atresia can be differentiated on MRI based on their location and signal intensities. In jejunal atresia, the obstruction occurs in the proximal small intestine, causing multiple dilated loops of the jejunum, which appear hypointense on T1 and hyperintense on T2 due to the fluid content. In contrast, ileal atresia occurs in the distal small intestine, resulting in dilated ileal loops that appear hyperintense on both T1 and T2 because of the presence of meconium or swallowed amniotic fluid. These MRI characteristics, along with the location of the obstruction, allow for a clear distinction between the two conditions, aiding in the diagnosis and management of fetal intestinal atresia [8].

#### Atresia with intestinal volvulus and meconial peritonitis

Atresia with intestinal volvulus and meconium peritonitis is often associated with fetal ascites, intestinal dilation, extraluminal calcifications, and polyhydramnios [9].

**Fig. 4:**
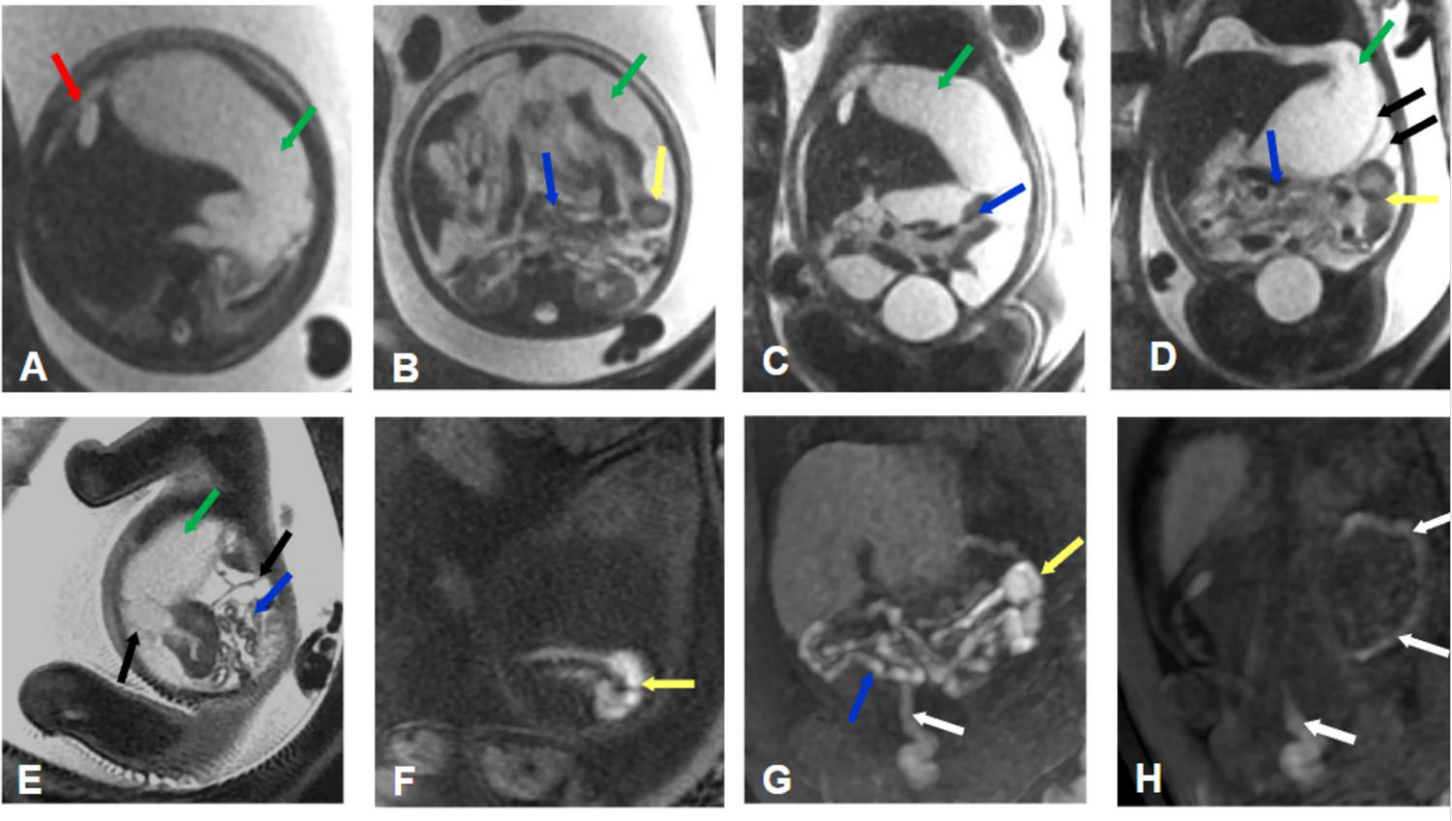
Atresia and intestinal volvulus with meconial peritonitis. Fetal ultrasound: ileal atresia and meconial peritonitis. Gestational age 32 weeks and 3 days. **A**, **B** Axial SS FSE T2. **C**, **D** Coronal SS FSE T2. **E** Sagittal SS FSE T2. **F** Sagittal 3D LAVA. **G**, **H** Coronal 3D LAVA. Tension ascites (green arrows) secondary to meconial peritonitis with septations (black arrows) and meconium pseudocyst in the falciform ligament (red arrows). Angulation and dilation of the proximal ileal loop in the left flank due to intestinal volvulus (yellow arrows), with the remaining bowel loops clustered without dilation (blue arrows). Secondary microcolon (white arrows)

While MRI has low sensitivity for detecting calcifications, it is highly effective in identifying dilated bowel loops, ascites, polyhydramnios, and cystic masses, such as meconium pseudocysts (Fig. 4). These pseudocysts may contain septations within them.

It is important to note that these prenatal findings can resolve over time, and intestinal perforation may heal without leading to long-term complications such as intestinal atresia or stenosis.

#### Cystic lymphatic malformation

Cystic lymphatic malformations include lymphatic cysts located in the mesentery, omentum, and retroperitoneum. These malformations typically appear in the second or early third trimester (19–31 weeks) of pregnancy.

Over time, the cysts tend to increase in size and are more commonly found on the left side of the abdomen. They can also change from unilocular to multilocular in structure. Due to adhesion to the mobile mesentery or omentum, the cysts may shift in position and can extend to the abdominal wall, thorax, or lower limb [10].

Differential considerations for these lesions include cystic neoplasms, abscesses, and other vascular malformations. Key distinguishing features on MRI include the lesion’s location, presence or absence of solid components, septal thickness, and enhancement patterns. For example, abscesses may show restricted diffusion and inflammatory changes, while cystic neoplasms usually have more solid and nodular components with irregular enhancement.

#### Cystic mesenchymal hamartoma

**Fig. 5:**
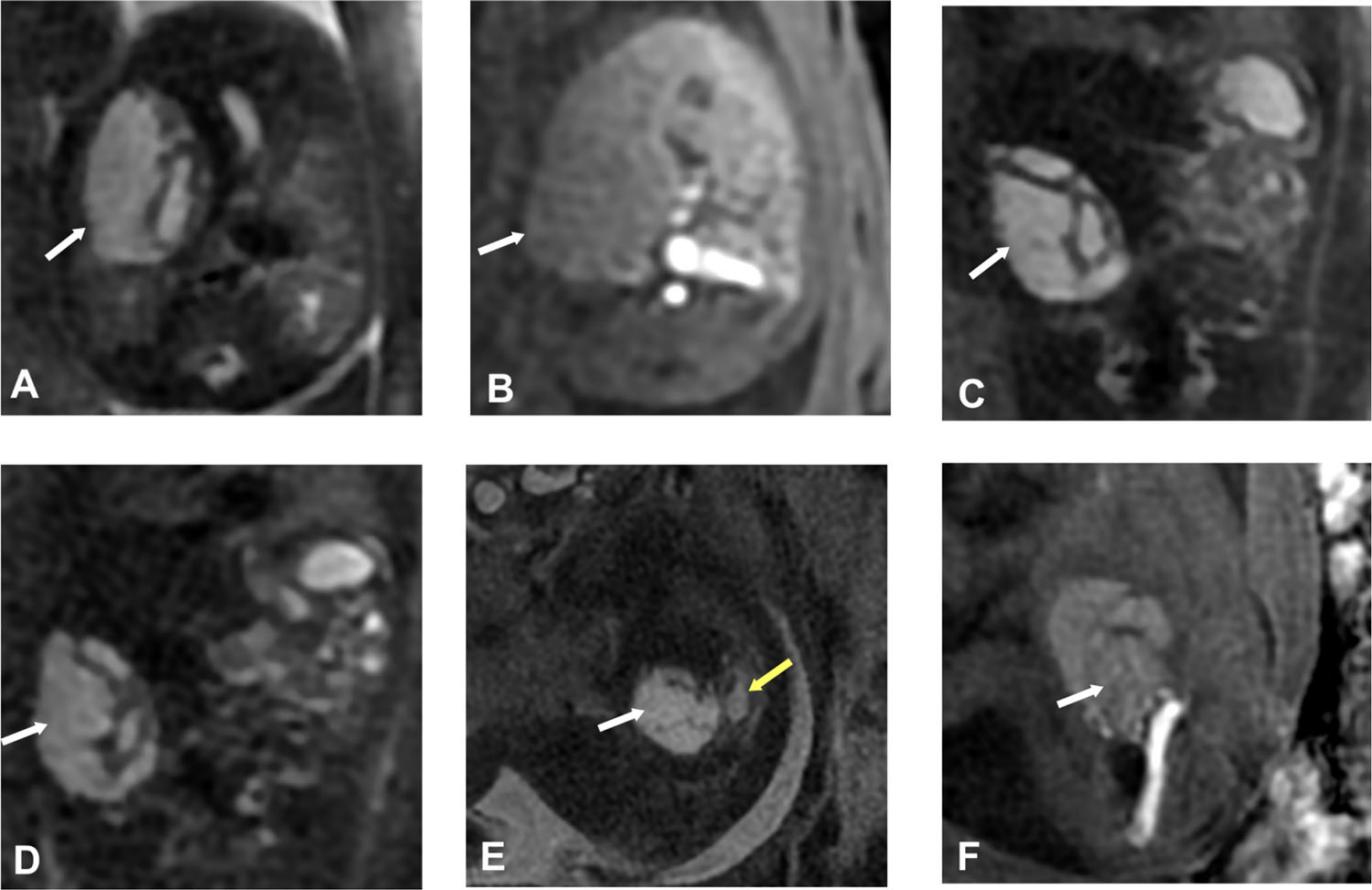
Hepatic mesenchymal hamartoma. Fetal ultrasound: septated hepatic cyst. Gestational age 21 weeks and 1 day. **A** Axial SS FSE T2. **B** Axial 3D LAVA. **C**, **D** Coronal SS FSE T2. **E** Sagittal FIESTA. **F** Sagittal 3D LAVA. Cystic mass (white arrows) in the right hepatic lobe, with septations inside, exophytic, isointense on T1 and hyperintense on T2, causing mild right renal ectasia (yellow arrow), suggesting mesenchymal hamartoma

Cystic mesenchymal hamartomas account for 23% of hepatic tumors diagnosed during the perinatal period. These tumors are more commonly located in the right lobe of the liver, and approximately 20% are pedunculated, which may cause them to appear extraluminal rather than intrahepatic (Fig. 5).

Several chromosomal translocations have been identified, suggesting that mesenchymal hamartomas could potentially be a form of neoplasia. The condition is thought to arise from various causes, including developmental issues like ductal plate malformation, local vascular injury or ischemia, or even as a response to toxic damage. Additionally, mesenchymal hamartoma are associated with placental mesenchymal stem villous hyperplasia, a type of vascular malformation, but are generally not linked to other congenital abnormalities [11].

The differential diagnosis of cystic mesenchymal hamartoma includes hepatic cysts, hemangioendothelioma, and cystic hepatoblastoma.

The prognosis for cystic mesenchymal hamartomas is generally worse when diagnosed in utero compared to postnatal diagnosis, with a reported mortality rate of 29%. However, there are cases of partial spontaneous regression, particularly in tumors with a prominent angiomatous component or embryonal carcinoma, offering some hope for recovery in certain cases [12].

#### Pancreatic cyst

Pancreatic cysts are more common in females and are typically located in the body and tail of the pancreas.

Pancreatic cysts are epithelial-lined cysts believed to result from abnormal development of the pancreatic ducts. This developmental anomaly may sequester secretory cells, leading to cyst formation. Prenatally, pancreatic cysts have been associated with Beckwith–Wiedemann syndrome, asphyxiating thoracic dysplasia, renal-hepatic-pancreatic dysplasia, and narrow thorax with short-limb dwarfism. Small cysts have also been observed in Short Rib Polydactyly syndrome type I. Postnatally, there is an association with polycystic kidney disease, anorectal malformations, and von Hippel–Lindau disease [11].

Pancreatic cysts can present as unilocular, multilocular, or may even transition from a unilocular cyst to a septated cyst. In some cases, these cysts may bleed internally.

The prognosis for fetal pancreatic cysts is generally good if they are not associated with other congenital anomalies.

The differential diagnosis of pancreatic cysts consists of enteric duplication cyst, renal cyst, hepatic cyst, biliary cyst, and adrenal cyst [11].

#### Choledochal cysts

Choledochal cysts are also more common in females, with a reported female-to-male ratio of 9:1 in Japanese literature and 3:1 in Western literature. Approximately 22% of cases have associated congenital heart defects [13].

Other associations include colonic atresia, duodenal atresia, imperforate anus, pancreas divisum, pancreatic aplasia, multi-septated gallbladder, aortic hypoplasia, congenital absence of the portal vein, heterotopic pancreatic tissue, OMENS-plus syndrome, familial adenomatous polyposis, and biliary atresia.

Regarding genetic alterations, the association between choledochal cysts and chromosomal duplication on 17q12 has been published [14]. In the article by Radana Kotalova et al, a case was presented in which a patient with a choledochal cyst was found to have a duplication in the 17q12 region, which is known to contain genes involved in biliary tract development. This genetic alteration may play a role in the formation of choledochal cysts, suggesting that duplication of this chromosomal region could predispose individuals to develop these congenital biliary anomalies.

Choledochal cysts appear on imaging as cystic or fusiform dilations of the biliary tree, typically at the porta hepatis and separate from the gallbladder. Ultrasound (US) is the initial modality of choice, showing a cystic structure with possible mild intrahepatic duct ectasia, and may reveal sludge, stones, or rarely a solid neoplasm. Magnetic Resonance Cholangiopancreatography (MRCP) offers superior anatomical delineation, aiding in pre-surgical planning and serving as a non-invasive alternative to Endoscopic Retrograde Cholangiopancreatography (ERCP) [15].

Choledochal cysts can be differentiated from other cystic masses if communication with the biliary system is demonstrated.

#### Splenic cysts

Splenic cysts are relatively uncommon and are typically unilocular. They are most often diagnosed in the third trimester of pregnancy. These cysts are usually small and frequently decrease in size or resolve naturally either in utero or within the first two years of life without causing symptoms.

The exact pathogenesis is not well-established, but several theories have been suggested, such as the migration of pluripotent cells into the spleen, leading to metaplasia, the inclusion of coelomic mesothelial cells undergoing squamous transformation, the invagination of peritoneal endothelial cells, and the enlargement of lymphatic vessels [16].

Complications such as torsion, hemorrhage, rupture, and infection have not been reported in the fetal or perinatal period, nor in children or adults with splenic cysts.

The differential diagnosis of splenic cysts encompasses pancreatic cyst, hepatic cyst, cystic lymphatic malformation, enteric duplication cyst, renal cyst, urinoma, urinary collecting system dilation, and adrenal cyst [11].

### Genitourinary cystic lesions

#### Ovarian cyst

**Fig. 6:**
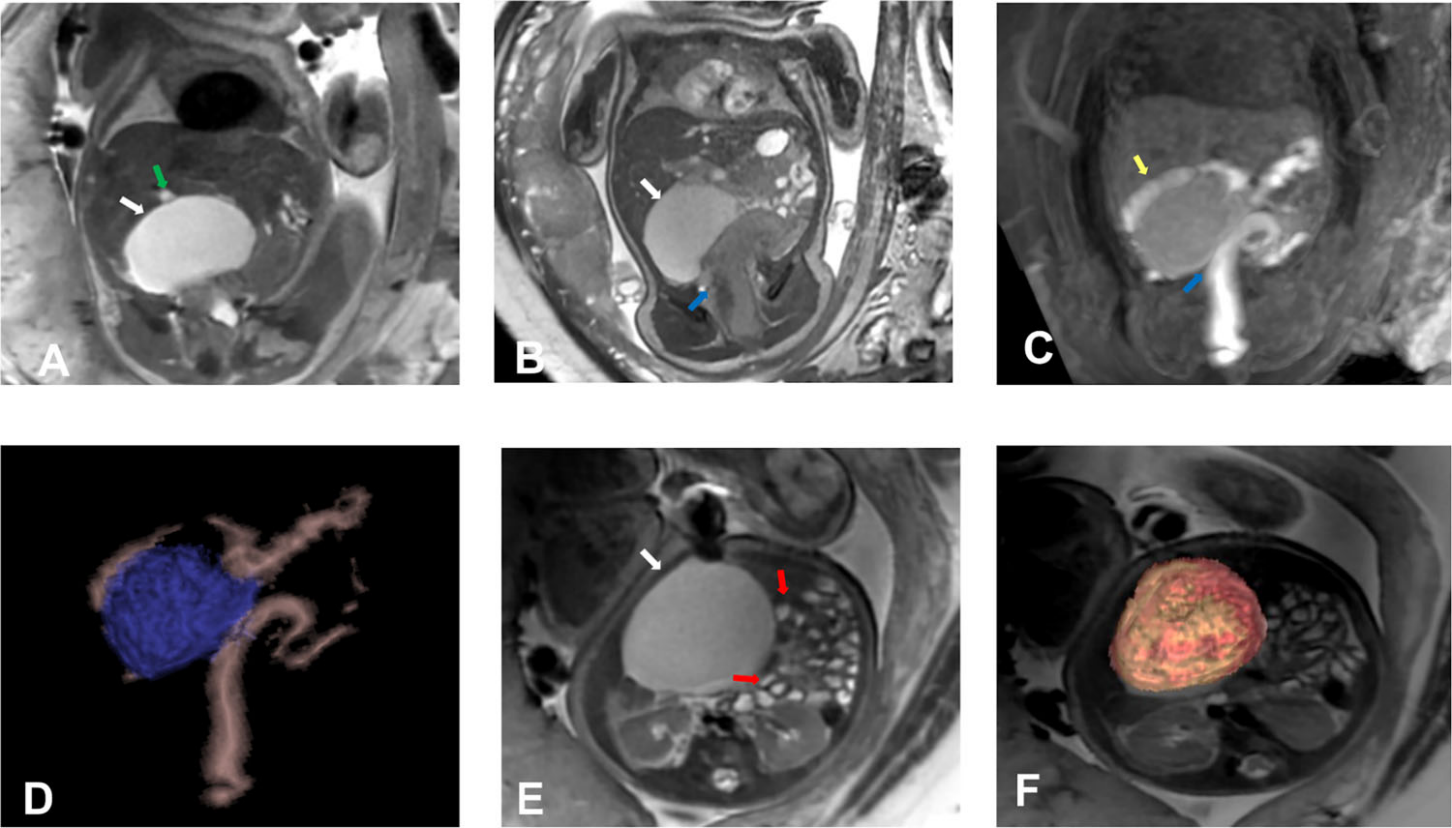
Right ovarian cyst. Fetal ultrasound: right hemorrhagic ovarian cyst. Gestational age 33 weeks and 3 days. **A** Coronal SS FSE T2. **B** Coronal FIESTA. **C** Coronal 3D LAVA. **D** 3D VR Coronal 3D LAVA. **E** Axial SS FSE T2. **F** 3D VR SSFSE. Intraperitoneal cystic lesion located in the right flank and right upper quadrant (white arrows) that slightly crosses the midline, indenting the hepatic flexure of the colon (yellow arrow), the inferior margin of the right hepatic lobe, and the gallbladder (green arrow). It displaces the loops of the small intestine to the left (red arrows) and contacts the distal sigmoid colon (blue arrows). It measures 53 mm in the transverse axis, 36 mm in the craniocaudal axis, and 48.5 mm in the anteroposterior axis

Prenatal ovarian cysts are relatively common, with an incidence of 1 in 2600 births. They are typically detected in the second or third trimester of pregnancy. These cysts are more often unilateral (Fig. 6), though they can be bilateral in 5% of cases.

During the fetal period, ovarian cysts can be simple or may become complicated by torsion, hemorrhage, and/or rupture [17]. Cysts ≥ 4 cm have a higher risk of spontaneous ovarian torsion.

Approximately 50% of ovarian cysts resolve during pregnancy or within the first few months after birth.

Polyhydramnios is observed in up to 18% of cases, likely due to extrinsic obstruction of the intestine by large cysts > 6 cm. In rare instances, severe mass effect may cause diaphragm elevation and lead to pulmonary hypoplasia [11].

#### Hydromucometrocolpos (hydrometrocolpos)

In fetal MRI, hydromucometrocolpos, also referred to as hydrometrocolpos, is characterized by a well-defined cystic dilation of the vagina and uterus due to fluid accumulation caused by distal obstruction, such as an imperforate hymen or vaginal atresia. On T2W images, these fluid-filled structures appear hyperintense, whereas on T1W images they are typically hypointense unless the fluid contains proteinaceous or mucoid material, which can increase T1 signal intensity. This condition may produce a mass effect, displacing adjacent pelvic organs and, in severe cases, compressing the bladder or ureters, potentially leading to urinary tract dilation [18].

Two main types are recognized [19]:

Secretory (hydrometrocolpos): this form arises secondary to obstructive pathology affecting the lower third of the vagina, uterus, or cervix, resulting in accumulation of intrauterine secretions stimulated by maternal estrogenic influence. The primary complication is ureterohydronephrosis due to compression of the distal ureters, sometimes followed by ascites. While most cases are sporadic, this type can be associated with genetic syndromes involving postaxial polydactyly, such as McKusick–Kaufman, Bardet–Biedl, and Pallister–Hall syndromes [20, 21], as well as with VACTERL and Mayer–Rokitansky–Küster–Hauser syndromes.

Urinary (urometrocolpos): this type is linked to congenital malformations of the urogenital system, including urogenital sinus or cloacal anomalies.

**Fig. 7:**
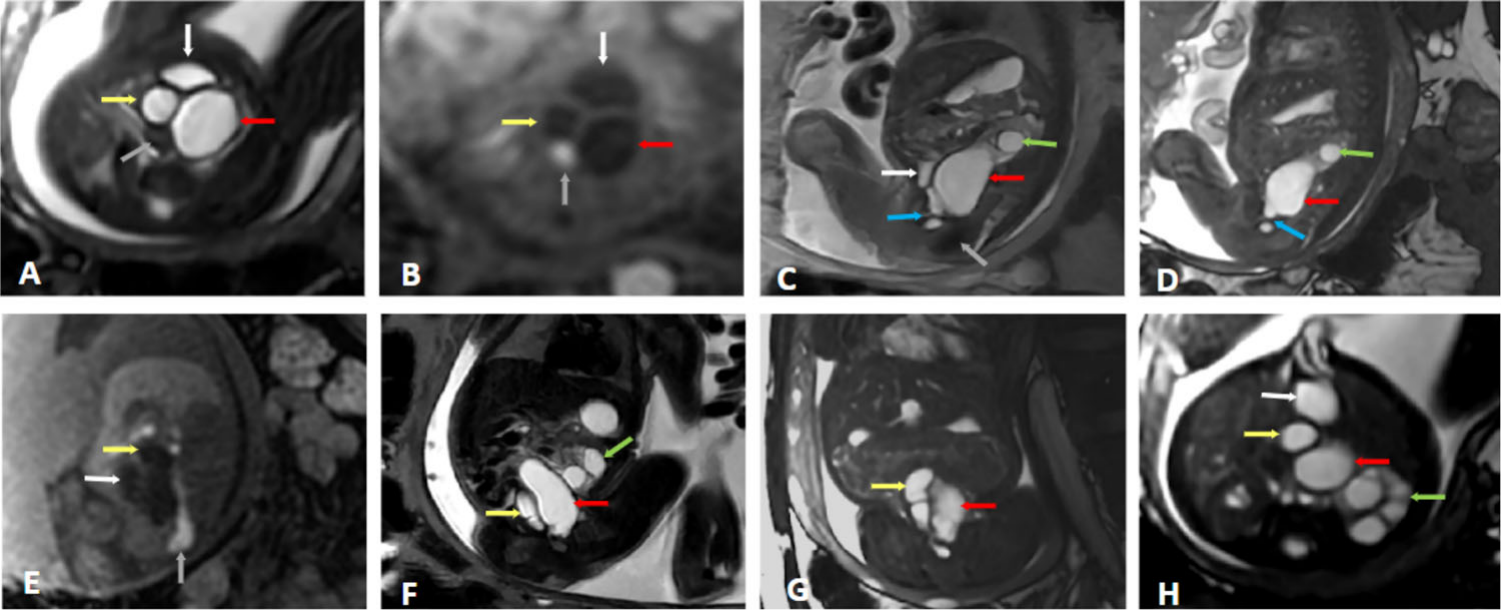
Urogenital sinus and hydrometrocolpos. Gestational age: 27 weeks and 1 day. MRI. **A** Axial SS FSE T2. **B** 3D Axial LAVA. **C** Sagittal SS FSE T2. **D** Sagittal FIESTA. **E** 3D Sagittal LAVA. **F** Coronal SS FSE T2. **G** Coronal FIESTA. **H** Axial FIESTA. Two tubular structures with a signal similar to the bladder are identified posterior to the bladder (white arrows) on both sides of the midline, communicating with the upper third of the vagina, corresponding to a didelphys uterus. The right hemi-uterus is located para-medially on the right, measuring 25.4 mm in the cranio–caudal axis (yellow arrows), and the left hemi-uterus, which is larger, is situated para-sagittally on the left, measuring 39 mm in the cranio–caudal axis (red arrows). Additionally, a transverse vaginal septum (blue arrows) is observed at the junction of the upper and middle third of the vagina, measuring 4.2 mm in the anteroposterior axis and 2.2 mm in the cranio–caudal axis, which causes vaginal obstruction. There is no communication with the rectum (gray arrows). The left kidney is dysplastic (orange arrows)

An imperforate hymen is the leading cause of hydrometrocolpos, affecting approximately 0.1% of term-born girls. Less common obstructive causes include vaginal stenosis or septa, vaginal atresia or hypoplasia, and cervical stenosis (Fig. 7).

A key imaging feature that aids in differential diagnosis is the presence of two cystic structures of different sizes—corresponding to the uterus and vagina—connected by a narrow tract representing the cervix. This creates the characteristic “fjord sign,” which helps distinguish hydrometrocolpos from ovarian cysts [22].

#### Cloacal malformation

Cloacal malformation is a rare and complex congenital anomaly that occurs in 1 in 50,000 live births, exclusively affecting female fetuses. It results from the failure of the cloaca to divide into the urogenital sinus and rectum, leading to a single external opening for the urinary, genital, and intestinal tracts.

The condition can present as either a dilated or stenotic cloaca, each with distinct complications. A dilated cloaca appears as a pelvic cystic mass, while a stenotic cloaca obstructs urinary flow, causing retrograde urine reflux into the vagina and uterus. In some cases, urine may reach the peritoneal cavity through the fallopian tubes, leading to urinary ascites [23].

Prenatal MRI plays a crucial role in diagnosing cloacal malformation by providing a detailed anatomical assessment. Typical findings include a pelvic cystic mass, dilated large intestine with increased T2 signal, absence of rectal visualization, and hydrocolpos, which is a common feature. Additionally, an increased T1 signal in the bladder may suggest the presence of meconium in the colon.

Given the variability of findings, MRI is essential for differentiating cloacal malformation PMID: 2from other pelvic anomalies and for planning early postnatal surgical intervention. This condition is often associated with VACTERL syndrome, ureteral ectopia, and duplications of the bladder or uterus. The presence of fetal ascites alongside a multiloculated pelvic cystic mass in a female fetus should raise a strong suspicion of this diagnosis [24].

Typical uterine anomalies associated with cloacal malformation include a bicornuate or didelphys uterus resulting from abnormal development and fusion of the Müllerian ducts. Other common abnormalities include uterine hypoplasia or agenesis, duplication of the uterine cavity, and malformations of the cervix, such as a septate or absent cervix. These anomalies arise due to embryological disruption of the genital tract development that often accompanies cloacal malformation and can impact reproductive function postnatally [25].

#### Congenital adrenal cyst

Simple adrenal cysts are often detected incidentally during routine imaging examinations. Their course is benign, and they may resolve spontaneously.

Certain syndromes, such as Beckwith–Wiedemann syndrome, include dysplastic cortical cysts, which are typically bilateral. These cysts have the potential to enlarge and hemorrhage [26].

It is remarkable the importance of cystic neuroblastoma as a differential diagnosis of a simple adrenal cyst, highlighting the utility of diffusion-weighted imaging.

**Fig. 8:**
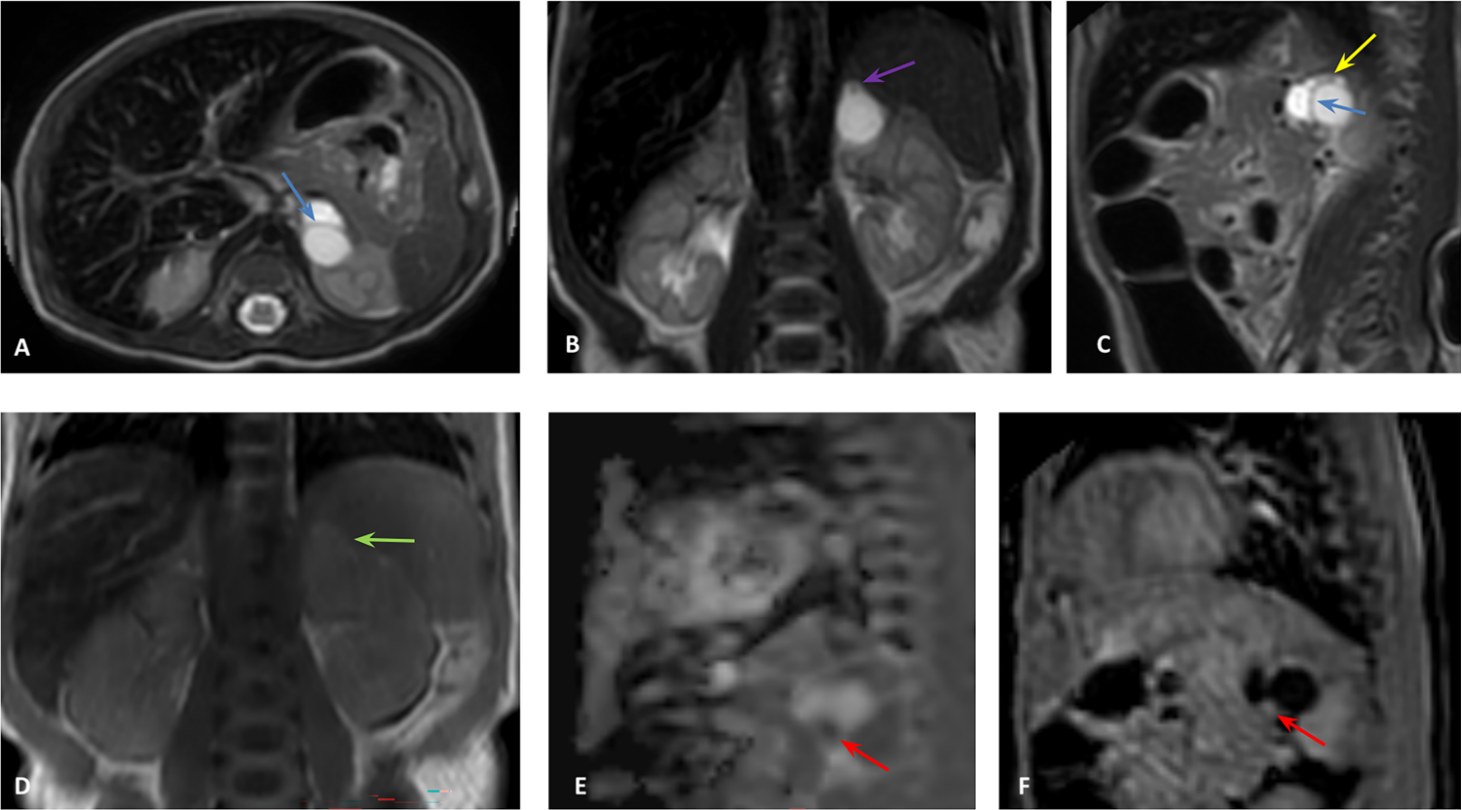
Left adrenal cystic neuroblastoma. **A** Axial FSE T2. **B** Coronal FSE T2. **C** Sagital FSE T2. **D** Postnatal Coronal T1. **E** Postnatal Sagital ADC. **F** Postnatal T1 +Gd. A hyperintense cystic lesion in the left adrenal gland is identified on T2 and hypointense on T1 (green arrow), measuring 13 × 17 × 14 mm (T × AP × L), with an internal septum (blue arrow) and thickening of the upper wall (yellow arrow). On diffusion sequence, two areas of restriction are observed, showing lower signal on T2 and enhancement after intravenous contrast administration: one at the upper pole (purple arrow on T2) and another nodule at the lower portion (red arrow on ADC and after contrast on T1 + Gd). The lesion exerts mass effect on the upper pole of the ipsilateral kidney. No signs of infiltration into neighboring structures are observed

Cystic adrenal neuroblastoma is an important differential diagnosis in the evaluation of suprarenal cystic lesions. Unlike simple adrenal cysts, which typically show homogeneous signal intensity on T2W images, thin walls, and no enhancement or diffusion restriction, cystic neuroblastomas often present with internal septations, thickened walls, solid nodular components, and areas of restricted diffusion on DWI/apparent diffusion coefficient (ADC) maps. These features suggest the presence of viable tumor tissue within the cystic mass. In the presented case (Fig. 8), the lesion demonstrates peripheral and nodular enhancement after gadolinium administration, and two foci of restricted diffusion, correlating with the solid enhancing areas, which support the diagnosis of cystic neuroblastoma rather than a simple adrenal cyst. DWI is especially helpful in distinguishing active tumor components from necrotic or cystic areas, thus playing a critical role in both diagnosis and treatment planning.

It is crucial to clarify that gadolinium contrast is not administered in utero. The enhancement seen in this case refers to the postnatal MRI [27].

#### Infradiaphragmatic bronchopulmonary sequestration

Infradiaphragmatic extralobar sequestration accounts for 10–15% of cases, with 75–90% occurring on the left side.

Bronchopulmonary sequestration appears as a T2-hyperintense solid lesion. Identification of the systemic vascular supply, particularly using balanced sequences, is key to diagnosis.

The differential diagnosis includes neuroblastoma and adrenal hemorrhage [28].

It should be noted that both pulmonary sequestration and neuroblastoma may demonstrate arterial vascular supply. However, diffusion-weighted imaging (DWI) can assist in distinguishing between them: pulmonary sequestration typically does not show diffusion restriction, whereas neuroblastoma often does [29].

#### Neuroblastoma

Neuroblastoma is the most common malignant congenital neoplasm. More than 90% of fetal cases originate in the adrenal gland, with a predominant right-sided location (60%).

Approximately 50% of these tumors are cystic, and cystic changes may indicate ongoing involution. Calcifications, a hallmark of pediatric neuroblastoma, are less frequent in fetal neuroblastoma.

Most fetal neuroblastomas exhibit a favorable DNA index (> 1) and lack Myc-N amplification.

Fetal neuroblastoma generally has a better prognosis, with potential spontaneous resolution in utero or shortly after birth [26].

In rare cases, fetal neuroblastomas may present with liver metastases or lead to hydrops fetalis.

In MRI, neuroblastoma appears as a heterogeneously enhancing solid mass that often crosses the midline and encases or displaces vessels. Both CT and MRI can demonstrate metastases to the liver, lymph nodes, bones, and skin. Whole-body MRI is particularly useful for staging and surveillance, as it provides superior sensitivity for detecting osseous metastases compared to CT and MIBG scintigraphy. However, its specificity remains low, as it can be challenging to distinguish between treated and active disease. MRI is especially beneficial for evaluating spinal involvement, as it provides detailed visualization of intraspinal extension, spinal cord compression, and perimedullary leptomeningeal space involvement [30].

#### Renal lesions

**Fig. 9:**
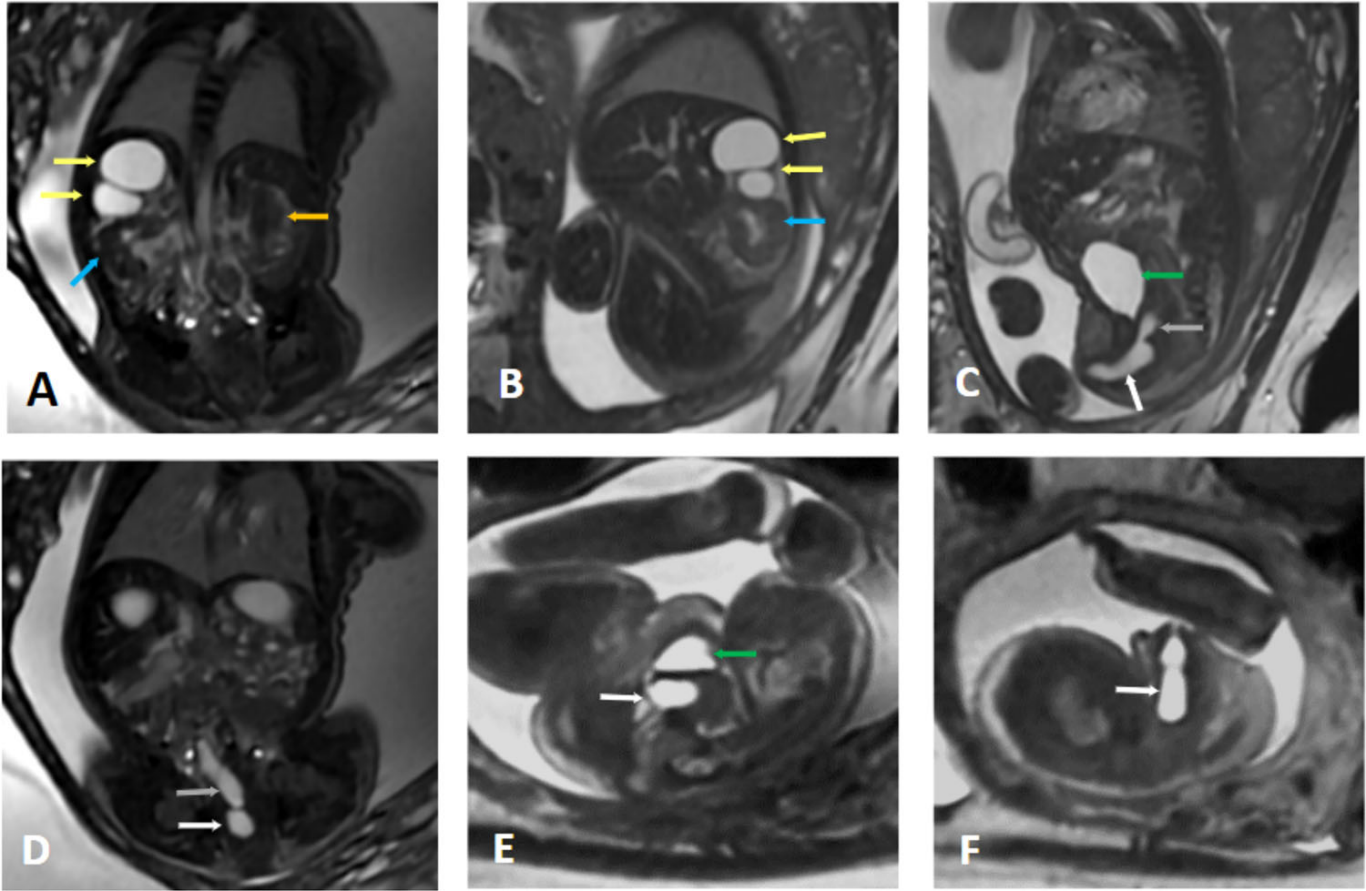
Right double pelvicalyceal system with ectopic ureter to the vagina. Gestational age: 28 weeks and 2 days. **A** Coronal SS FSE T2. **B**, **C** Axial FIESTA. **D** Coronal FIESTA. **E**, **F** Axial SS FSE T2. Right double pelvicalyceal system with cystic dilatations of the upper pelvis (yellow arrows). The lower right pelvis is not dilated (blue arrows). Superior right ectopic ureter (gray arrows) draining into the vagina (white arrows). Normal left kidney (orange arrow) and bladder (green arrows)

- Multicystic dysplastic kidney: often associated with other anomalies such as ureteropelvic junction stenosis or vesicoureteral junction stenosis. Although most cases are sporadic, a genetic cause has been identified in some instances. Mutations in the PAX2, TCF2 (HNF beta), and uroplakin genes have been recognized [26].
- Hydronephrosis: commonly secondary to ureteropelvic junction stenosis or a double pelvicalyceal system (Fig. 9), which results in dilation of the upper pelvis and/or ureterocele.

#### Primary megaureter

Occurs bilaterally in 20% of cases, and in 5% of cases, there are contralateral genitourinary anomalies. Primary megaureter is classified into three subtypes: obstructive, with reflux, and without reflux or obstruction, with the latter being the most common [31].

#### Bladder and/or ureteral dilation

- Posterior urethral valves: affect males, leading to obstruction of the urinary bladder outlet. Imaging typically reveals distension of the posterior urethra, known as the keyhole sign, along with dilation and thickening of the bladder wall, with or without ureteral dilation [26].
- Prune-belly syndrome: also known as Eagle–Barrett syndrome, occurs almost exclusively in males. This condition is characterized by cryptorchidism (undescended testicles), urinary tract anomalies such as renal hypoplasia and dysplasia, ureteral dilation with thick walls, megacystis, dilation of the prostatic urethra, prostatic hypoplasia, and abnormally lax and deficient abdominal wall musculature. Associated findings include abdominal distension and a small thorax with hypoplastic lungs. Amniotic fluid may be normal or decreased in these cases [26].
- Megacystis-microcolon-intestinal hypoperistalsis syndrome: is a rare autosomal recessive congenital disorder that primarily affects females (70%) and is often lethal within the first year of life. This condition causes severe abdominal distension due to a markedly dilated urinary bladder, but without obstruction, due to absent or abnormal bladder wall motility. Additionally, colonic and small bowel peristalsis is nearly absent. It is suspected that a megacystis is identified in a female fetus without an obvious urological etiology, especially when there is the absence of ureterocele, midgut malrotation, and a positive family history [26].

#### Urachal anomalies

The most common urachal anomalies include urachal cysts (45%), urachal sinus (37%), and patent urachus (16%). Urachal diverticula are extremely rare. Urachal cysts typically present as midline anterior cysts, located cranial to the bladder, with no significant changes in size or shape over time [32].

#### Allantoic cysts

They occur in the context of a persistent urachus, and appear as cysts near the anterior abdominal wall. A specific variant, the vesico-allantoic cyst, is located at the base of the umbilical cord and communicates with the bladder dome, leading to the separation of the umbilical cord vessels. Some of these cysts can grow very large or cause diffuse umbilical cord edema [33].

#### Omphalomesenteric cysts

These represent true umbilical cord cysts located proximally, without involvement of the cord vessels or communication with the bladder dome. They are frequently associated with abdominal wall defects and the presence of Meckel’s diverticulum [23].

#### Renal urinoma

A renal urinoma refers to a fluid collection resulting from urinary extravasation, commonly secondary to urinary tract obstruction, trauma, or congenital anomalies.

It is often associated with conditions like ureteropelvic junction obstruction (UPJO), posterior urethral valves (PUV), or other congenital anomalies affecting urinary drainage.

**Fig. 10:**
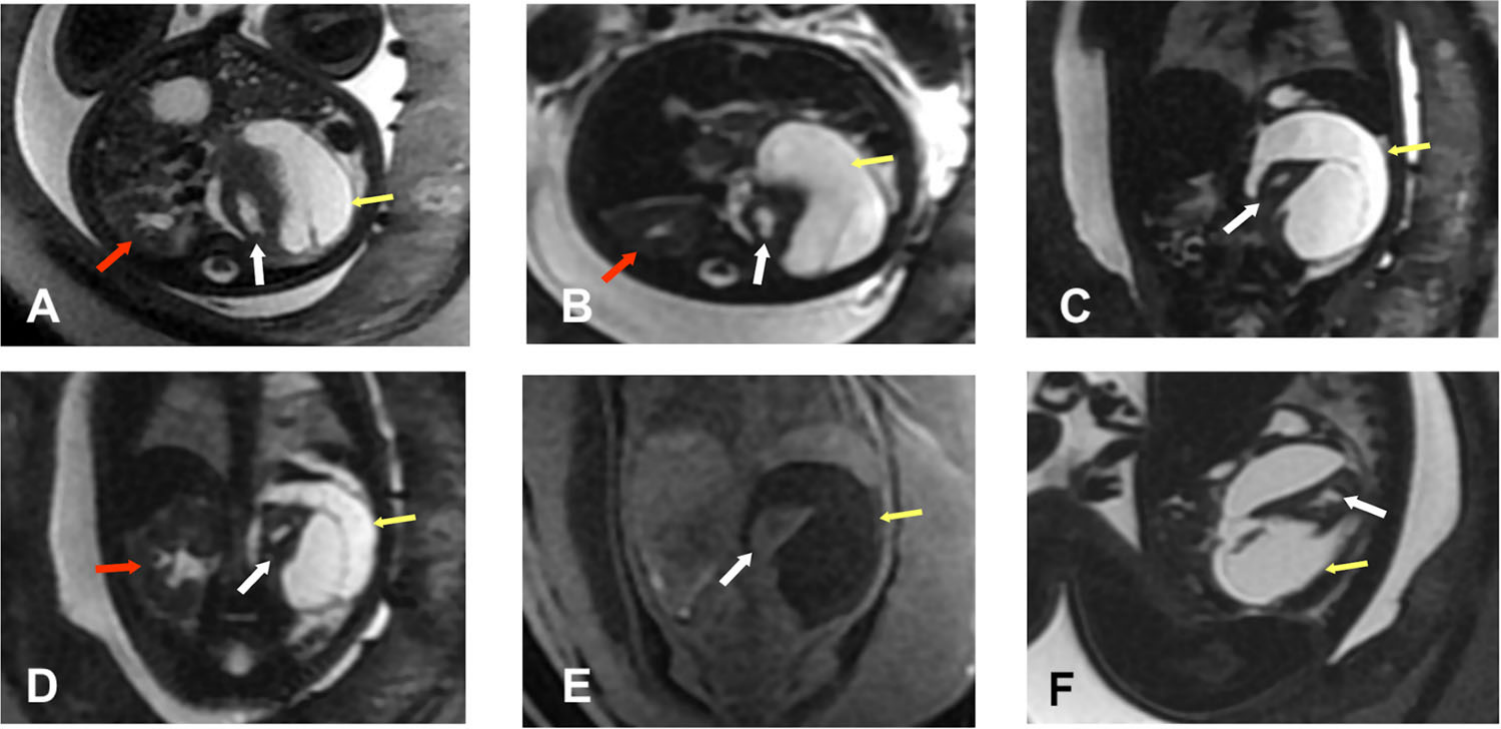
Left perirenal urinoma. Fetal ultrasound: perirenal hemorrhage or hemorrhagic cyst. **A**, **B** Axial SS FSE T2. **C**, **D** Coronal SS FSE T2. **E** Coronal 3D LAVA. **F** Sagittal SS FSE T2. Normal right kidney (red arrows). Perirenal cystic lesion (yellow arrows) surrounding the left kidney (white arrows), compressing and displacing it anteromedially. It has septations inside without signs of bleeding in the T1 sequence. There are no signs of hydronephrosis. The MRI is reported as lymphangioma or retroperitoneal teratoma. The lesion progressively disappeared after birth and corresponded to a urinoma

Typically, a urinoma appears as a well-defined, fluid-filled mass adjacent to the affected kidney, often causing displacement or compression of renal parenchyma (Fig. 10). The signal characteristics of the urinoma on MRI are similar to those of urine, appearing hypointense on T1W images and hyperintense on T2W images. This imaging modality is particularly valuable when ultrasound findings are inconclusive or when a comprehensive evaluation of the urinary system is required [34].

### Teratomous and syndromic cystic lesions-sacrococcygeal teratoma

The sacrococcygeal teratoma is the most common tumor found in newborns. The high mortality rate associated with this tumor is primarily due to its size, which can lead to dystocia, premature birth secondary to polyhydramnios, and placentomegaly resulting from heart failure due to an arteriovenous shunt.

The Altman classification categorizes sacrococcygeal teratomas into four distinct types based on their anatomical location and extent.

**Fig. 11:**
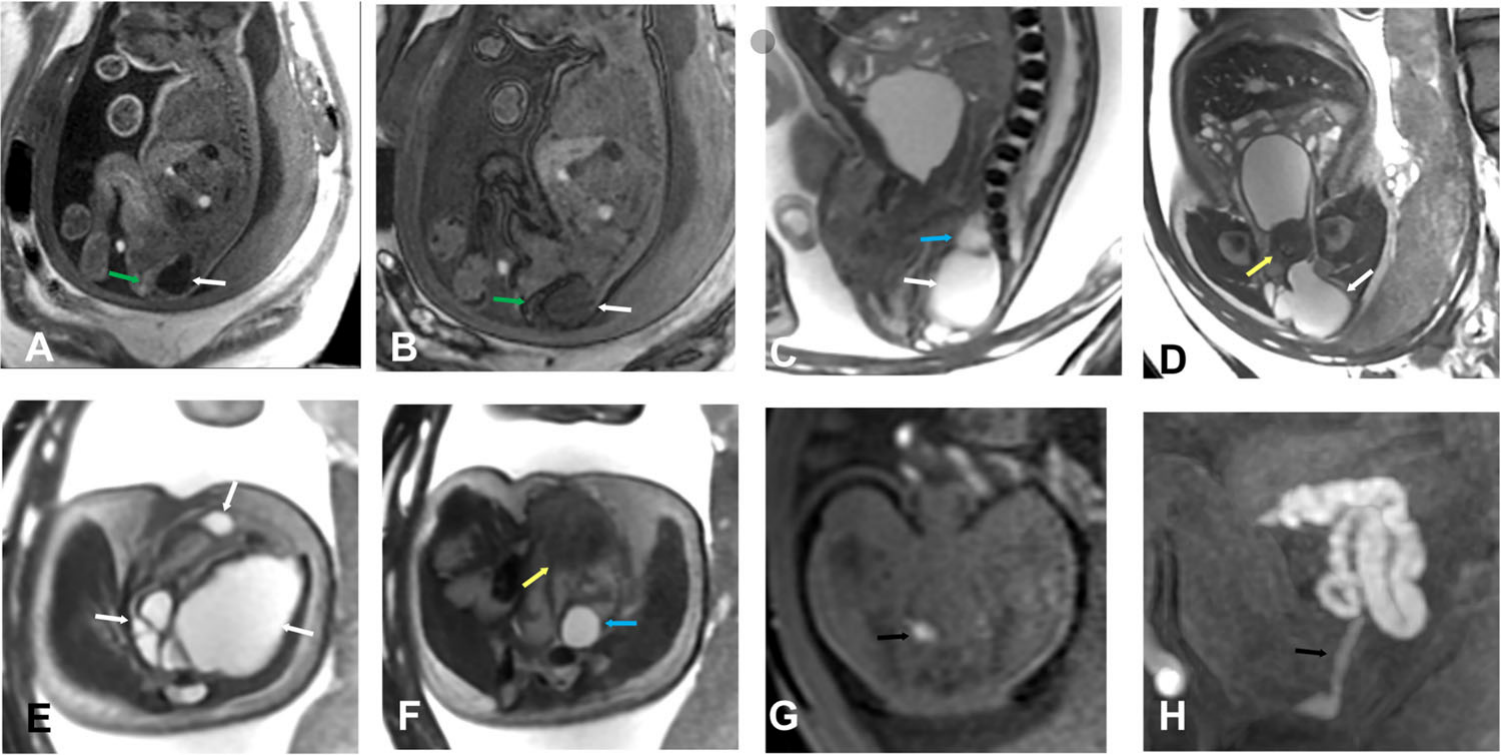
Type II sacrococcygeal teratoma. Gestational age: 33 weeks and 2 days. **A** Sagittal 3D LAVA in phase. **B** Sagittal 3D LAVA out of phase. **C** Sagittal FIESTA. **D** Coronal FIESTA. **E**, **F** Axial FIESTA. **G** Axial 3D LAVA. **H** Coronal MIP 3D LAVA. Cystic predominant mass (white arrows) originating from the sacrococcygeal region, affecting the gluteal and perineal regions bilaterally with left predominance and partially occupying the pelvis. It has a fatty component (green arrows) and a solid component (blue arrows). It indents the rectum (black arrows)
- Type III: minimal external component with a predominantly intrapelvic component.
- Type IV: completely located within the pelvis and abdomen.

- Type I: external component with a minimal presacral component.
- Type II: external component with a presacral component (Fig. 11).

In prenatal MRI, sacrococcygeal teratomas typically appear as well-defined, heterogeneous masses arising from the sacrococcygeal region. They may contain solid, cystic, and sometimes calcified components, leading to variable signal intensities on T1W and T2W images. Cystic areas appear hyperintense on T2 and hypointense on T1, while solid components may enhance with contrast. Large tumors can cause mass effect, displacing adjacent structures, and may be associated with vascularity, leading to potential complications like hydrops fetalis. MRI helps assess tumor composition, vascular involvement, and potential fetal compromise [35].

#### Currarino triad

**Fig. 12:**
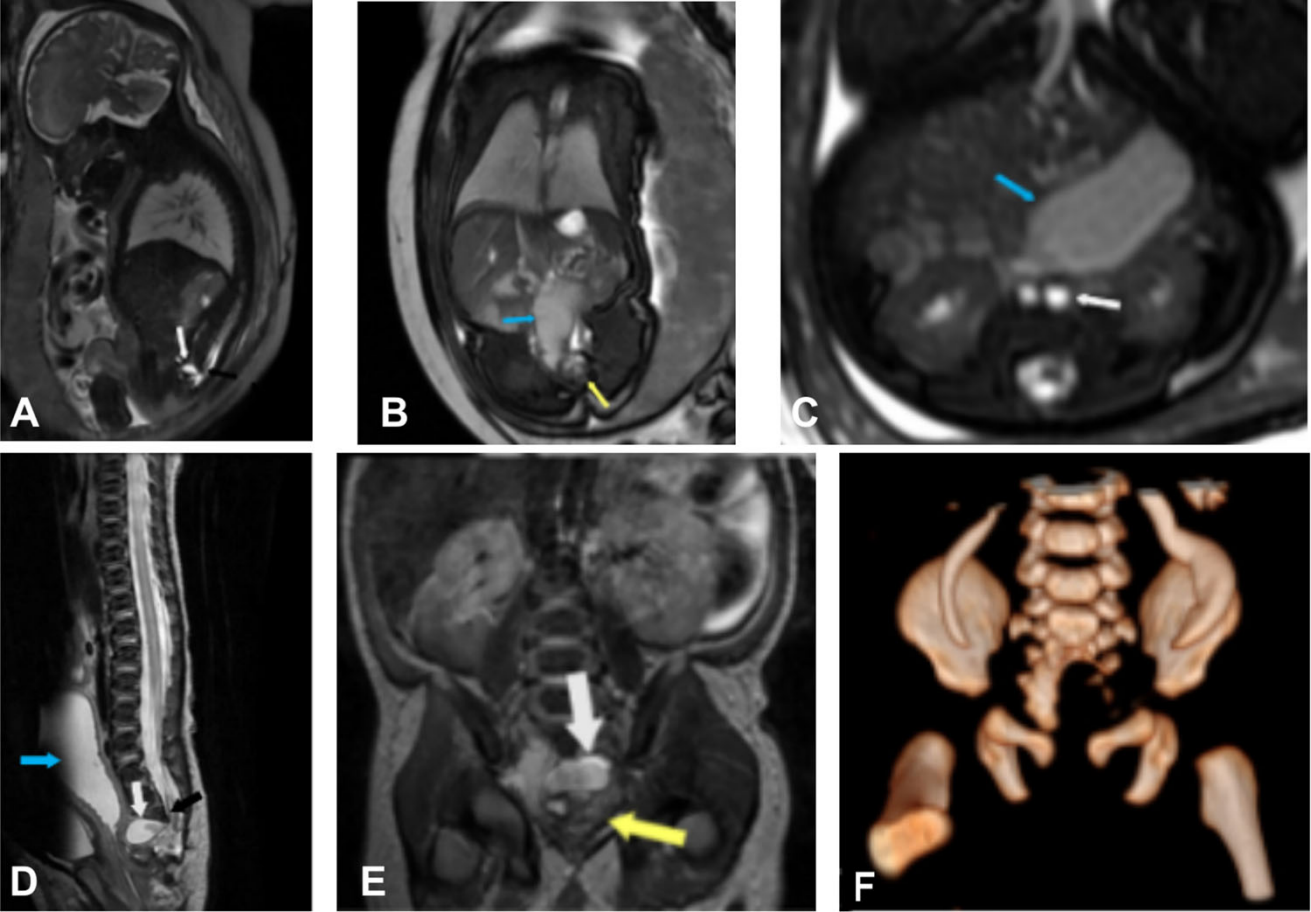
Currarino Triad. Gestational age: 33 weeks and 5 days. **A** Sagittal SS FSE T2. **B** Coronal FIESTA. **C** Axial FIESTA. Partial agenesis of the sacrum (black arrows), rectal dilation due to anorectal stenosis (blue arrows), anterior meningocele (white arrows), and presacral teratoma (yellow arrows). Postnatal control at 5 days of life (**D**) Sagittal FSE T2. **E** Coronal FSE T2. **F** VR reconstruction CT

In 1981, Currarino et al described a clinical triad, now known as Currarino Syndrome, characterized by the following three components (Fig. 12):

1. A sacrococcygeal defect, which can range from asymmetrical deformities, such as a scimitar-shaped or sickle-shaped hemisegment, to total sacral agenesis.
2. A presacral mass, with anterior meningocele being the most common, although other lesions such as teratomas, enteric cysts, dermoid or epidermoid cysts, lipomas, hamartomas, or rectal duplications may also be present.
3. Anorectal malformations, including conditions like anal stenosis (with or without a fistula), atresia, ectopic anus, imperforate anus, and cloaca.

In addition to the triad, Currarino Syndrome can be associated with other manifestations, including urogenital system malformations such as horseshoe kidneys, single pelvic kidneys, neurogenic bladders, multicystic kidneys, vesicoureteral reflux, and duplication of the vagina, clitoris, or uterus [36].

Furthermore, intraspinal anomalies are frequently observed, which may include a low-lying tethered spinal cord, hydromyelia, intraspinal lipomas, and hydrocephalus.

Sacral agenesis is a crucial diagnostic element and can range from total sacral agenesis to partial agenesis or hemisacrum. Presacral masses, commonly anterior meningoceles and teratomas, are detected on MRI. Meningoceles appear hypointense on T1W images and hyperintense on T2W images, with no enhancement after gadolinium administration. Teratomas appear as heterogeneous cystic masses. Additionally, anorectal anomalies, such as anorectal stenosis, are observed, although not directly assessed by MRI. MRI plays a critical role in evaluating these conditions, offering detailed imaging of sacral defects and associated presacral masses, which helps in diagnosing CS [37].

## Conclusion

Fetal MRI significantly enhances the diagnostic accuracy of abdominal cystic lesions detected on prenatal ultrasound. Its multiplanar capabilities and superior soft tissue characterization allow for precise lesion classification, aiding in the differentiation of gastrointestinal, genitourinary, teratomatous, and syndromic conditions such as Currarino’s triad. By providing detailed anatomical information, fetal MRI plays a crucial role in refining prenatal diagnosis, optimizing perinatal care, and guiding postnatal surgical strategies. The integration of fetal MRI into routine prenatal evaluations can improve clinical decision-making, reduce diagnostic uncertainty, and ultimately enhance neonatal outcomes.

## Data Availability

The datasets generated and/or analyzed during the current study are not publicly available due to patient confidentiality, but are available from the corresponding author on reasonable request.

## Abbreviations

ADC: Apparent diffusion coefficient
CT: Computed tomography
DWI: Diffusion weighted imaging
FIESTA: Fast imaging employing steady-state acquisition
MRI: Magnetic resonance imaging
SSFSE: Single-shot fast spin echo
T1W: T1-weighted sequences
T2W: T2-weighted sequences
